# Effectiveness of a training programme to improve hand hygiene compliance in primary healthcare

**DOI:** 10.1186/1471-2458-9-469

**Published:** 2009-12-16

**Authors:** Carmen Martín-Madrazo, Asunción Cañada-Dorado, Miguel Angel Salinero- Fort, Juan Carlos Abanades-Herranz, Rosa Arnal-Selfa, Inmaculada García-Ferradal, Flora Espejo-Matorral, Enrique Carrillo-de Santa-Pau, Sonia Soto-Diaz

**Affiliations:** 1 Unidad de Formación e Investigación Area 4, Madrid, Spain; 2 Unidad de Calidad y Gestion de Riesgos Sanitarios Area 4, Madrid, Spain; 3 Unidad de Formación e Investigación Area 4, Madrid, Spain; 4 Gerencia Atencion Primaria, Area 4, Madrid, Spain; 5 Unidad de Formación e Investigación Area 4, Madrid, Spain

## Abstract

**Background:**

Hand hygiene is the most effective measure for preventing infections related to healthcare, and its impact on the reduction of these infections is estimated at 50%. Non-compliance has been highlighted in several studies in hospitals, although none have been carried out in primary healthcare.

**Main objective:**

To evaluated the effect of a "Hand Hygiene for the reduction of healthcare-associated infections" training program for primary healthcare workers, measured by variation from correct hand hygiene compliance, according to regulatory and specific criteria, 6 months after the baseline, in the intervention group (group receiving a training program) and in the control group (a usual clinical practice).

**Secondary objectives:**

-To describe knowledges, attitudes and behaviors as regards hand hygiene among the professionals, and their possible association with "professional burnout", stratifying the results by type of group (intervention and usual clinical practice).

-To estimate the logistic regression model that best explains hand hygiene compliance.

**Methods/Design:**

Experimental study of parallel groups, with a control group, and random assignment by Health Center.

Area of study.- Health centers in north-eastern Madrid (Spain).

Sample studied.- Healthcare workers (physicians, odontostomatologists, pediatricians, nurses, dental hygienists, midwife and nursing auxiliaries).

Intervention.- A hand hygiene training program, including a theoretical-practical workshop, provision of alcohol-based solutions and a reminder strategy in the workplace.

Other variables: sociodemographic and professional knowledges, attitudes, and behaviors with regard to hand hygiene.

Statistical Analysis: descriptive and inferential, using multivariate methods (covariance analysis and logistic regression).

**Discussion:**

This study will provide valuable information on the prevalence of hand hygiene non-compliance, and improve healthcare.

## Background

Infections related to healthcare are among the most important causes of morbidity and mortality in hospitalized patients. A study of prevalence carried out by the World Health Organization (WHO) in 55 hospitals from 14 countries, showed that 8.7% of hospitalized patients contract Nosocomial Infections (NI). The importance of NI in terms of morbidity, mortality, impact on quality of life in patients and relatives and secondary economic costs, has been emphasized repeatedly in the last years [1]. In the developed countries, around 5-10% of patients admitted to hospitals for acute conditions presented an infection that was not being incubated or present at the time of admission. Healthcare-related infections are the direct cause of 80,000 deaths in the United States and 5,000 deaths in England every year [2,3]. According to data from the Survey on Prevalence of Nosocomial Infection in Spain (EPINE study) for 2006 [4], NI affected between 7% and 9% of patients admitted to Spanish hospitals. These data are very similar to those for developed countries in terms of frequency, economic cost and mortality [5]. NI present many of the characteristics that define a significant problem in patient safety: affect millions of people all over the world, complicate patient care, contribute to the patient death or temporary/permanent disability, increase resistance to antimicrobials and generate substantial additional costs in the treatment of the patient disease.

There are many causes of NI, which are related to healthcare systems and processes, as with the behavior of the professionals involved. The results of the Study of the Efficacy of Nosocomial Infection Control (SENIC study) finding that vigilance is an effective method for the prevention of NI [6,7]. Indeed, in the hospitals included in the infection prevention program where prevention and control activities were carried out, infection rates was a reduction near to 32%. Other studies have shown the benefits of NI prevention in healthcare and economic terms [8,9].

### Measures to reduce infections related to healthcare: hand hygiene

The areas of action against these infections are based on simple and well established precautions which have been seen to be effective and widely accepted - the "ordinary precautions" cover all the basic principles for controlling infections that required in all healthcare centers. They are applied to all patients regardless of their diagnosis, risk factors, and infection status, in order to reduce the risk to the patient and healthcare workers of contracting infections. Hand hygiene (HH) is an important element in ordinary precautions and is the most effective measure for preventing infections.

The hands of health workers (HCWs) are the most common carrier of transmission of microorganisms from one patient to another, from one area of the patient's body to another and from a polluted environment to patients. The HH is considered the most important measure, because of its proven efficiency (it is estimated that the impact on the reduction of NI is 50%), its effectiveness, and its low cost [10]. However, there is poor compliance with HH regulations by healthcare workers all over the world, and all the studies carried out in hospitals suggest that the frequency of compliance is lower than 50% of the opportunities in which the practice is considered a priority [11,12].

There are different factors contributing to low levels of HH compliance, both among the professionals: lack of knowledge of the importance of preventing NI, a lack of understanding of the appropriate techniques involved, the occurrence of contact dermatitis; and by the healthcare organization: staff shortaged, work overload, difficult access to points used for conventional hand hygiene, and finally, the absence of an institutional commitment to overall improvement of HH.

Pittet et al [13], carried out a study in a university hospital, based on direct observation of physicians, and identified behavioral factors associated with beliefs, attitudes and perceptions in non-compliance of HH. There was over 75% believed that not performing HH led to a higher risk of cross-transmission, 72% thought that HH was unnecessary after removing gloves and 72% thought that HH was necessary after each patient.

In an epidemiological study of HH carried out in 1994 in hospitals affiliated to the University of Geneva, was observed an average rate of compliance of 48% [14]. This study identified as factors associated with a lack of compliance: professional category (nurses had higher rates of compliance than other professionals), high risk activities for NI in units caring for patients in a critical condition, undertaking procedures with a high level of bacterial contamination, and an overload of work among healthcare professionals.

In another study, in a Spanish hospital [15], about HH compliance and its associated factors, the average for compliance was 31%. This is very low, regarding that the observation was made after a period of health education on the HH and with the prior knowledge of the professional that they were being evaluated.

The Atlanta Centers for Disease Control and Prevention (CDC) published an extensive review of recommendations for HH in healthcare institutions in 2002. It recommended using alcohol-based solutions, instead of washing hands with soap or antiseptic, in order to increase compliance with this action for the prevention of NI [16].

Numerous studies have shown that educational programs can effectively increase knowledges, positive attitudes and appropriate practice to ensure compliance with international protocols and regulations for the prevention and control of NI [17-19].

The Cochrane review in 2007 on "Interventions to improve hand hygiene compliance in patient care" concludes that there are few evidence to inform the choice of interventions to improve HH, and that studies with consistent designs are urgently required in order to examine the effectiveness of well designed interventions to increase HH compliance, and take into account the factors related to the behavior of HCWs, based on knowledge of the behavioral and social sciences [20].

The WHO in 2004, approved the creation of an "Alliance for Patient Safety", which acknowledged the universal need to improve HH in healthcare institutions, developing a strategy with a very clear call to action: "Clear hands are safer hands". These globally approved recommendations reinforce the need for multidisciplinary interventions, including important elements such as education and motivation on healthcare workers, the inclusion of alcohol-based solutions, the use of compliance indicators and a strong commitment by all healthcare managers [21,22].

The Quality Plan for the Spanish National Health System of 2007, in patient safety, proposed the development of strategies, measures and programs to promote safe practices in healthcare centers. These included the promotion of clean hands practices in all healthcare centers, and recommended the use of alcohol-based solutions as an effective measure to reduce the incidence of healthcare-associated infection [23].

In view of all the above, it seems necesary to carry out studys like the one proposed here, in order to evalued the effect of a hand hygiene training program (TP) on the reduction of healthcare-associated infections among health workers in primary healthcare centers, and to know what factors (intrinsic and extrinsic) are related with the failure (or inadequate compliance with) in the HH.

## Objectives

### Main Objective

-To evaluated the effect of a (TP) on "Hand Hygiene for the reduction of healthcare-associated infections" among primary healthcare workers, as measured by the variation from correct HH compliance by explicit regulatory criteria, 6 months after the baseline, in the intervention group and in the control group.

### Secondary objectives

-To describe knowledges, attitudes and behaviors regarding HH, and their possible association with professional burnout among the professionals evaluated, stratifying the results by type of Group (intervention and usual clinical practice).

-To estimate the logistic regression model that best explains HH compliance, where the main explanatory variable is the type of intervention and the covariables for which it will be adjusted: age, sex, type of profession, type of employment contract, years of employment experience, level of knowledge of HH, attitude to HH, behavior with regard to HH, and professional burnout.

## Methods/Design

### Study design

An experimental study of parallel groups, with a control group and random assignment intervencion by health centers.

**Figure 1:**
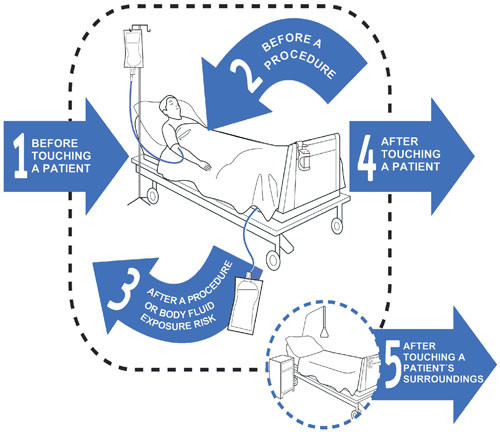
**Five moments of hand hygiene (reproduced with WHO permission)**.

Definition of terms:

-***Five Indications/moments ***are based on those defined by the WHO Guidelines on HH 20] (Figure 1).

A *Moment *is when there is a perceived or actual risk of pathogen transmission from

one surface to another via the hands. Healthcare workers hands will come in contact with many different types of surfaces while undertaking a succession of tasks.

The 5 Moments for HH are:

**Moment 1**: Before touching a patient

**Moment 2**: Before a procedure

**Moment 3**: After a procedure or body fluid exposure risk

**Moment 4**: After touching a patient

**Moment 5**: After touching a patient surroundings

#### 1) Before touching a patient

To protect the patient against acquiring harmful germs from the hands of the healthcare workers (e.g. taking arterial pressure, thorax auscultation, abdominal palpation).

#### 2) Before clean/aseptic procedures

To protect the patient from harmful germs (including their own) from entering their body during a procedure. Immediately before carrying out any task involving direct or indirect contact with mucous, damaged skin, an invasive medical device (e.g. a probe or catheter), preparation of medication (e.g. treatment of wounds, administration of eye drops, application of injectable materials, oral treatment).

#### 3) After a procedure or body fluid exposure risk

To protect yourself and the healthcare surroundings from harmful patient germs. If the professional uses gloves to carry out the task that involves a risk, he/she must remove them after completing the task to immediately carry out HH (e.g. extracting and handling any liquid sample, cleaning contaminated material, vomit).

#### 4) After touching a patient

To protect yourself and the healthcare surroundings from harmful patient germs (e.g. shaking hands, taking the pulse, thorax auscultation).

#### 5) After touching patient surroundings

To protect yourself and the healthcare surroundings from harmful patient germs (e.g. after calibrating a glucose meter, after teaching the patient how to use an inhaler).

The moments for HH are independent of those justifying the use of gloves. This means that the use of gloves does not in any way change the moments for HH and above all, does not replace HH.

##### -Action

This is recognition of the instructions by healthcare workers during their work. When the action is carried out (positive action) it can be done in two ways: by washing hands with an alcoholic disinfectant or by washing them with soap and water. The absence of the action (negative action) is considered as such when prior instructions have been given to carry out an action which has not occurred.

### Instruments of measurement

#### -Structured Observation (SO)

Each professional selected will be evaluated by direct observation, non-participating and structured, by a neutral professional with prior training, who is familiar with the concept of the WHO (Five) Well-being Index. The SO will be carried out at two moments: at the baseline and 6 months after the first.

##### Observer training

Through practical examples of the 5 moments and watching the WHO video on HH. After training, the level of concordance between their HH compliance criterion and that of a Group of Experts will be measured by a pilot test of 20 observations in a health center. If the Kappa Index (a test of agreement beyond chance) is greater than or equal to 80%, the observer receives approval to start the study. Otherwise, the training period is extended and concordance

re-evaluated after other practical examples.

The observer will collect the data in each observation in a data collection notebook, where he/she will record: the health center, type of profession, type of contract, years of employment experience, date of birth, and the positive or negative action involved in each moment.

### Efficiency variables

-HH compliance by each professional:

-Variation in the professional HH compliance:

This will be calculated by subtracting the 1st observation (baseline) HH compliance from the second (6 months later) by each professional. A positive difference shows an increase in HH compliance, and a negative result shows a decrease in HH compliance.

### Social and occupational variables

- Age, sex, profession, type of contract and years of employment experience.

### -Other variables

#### Questionnaire on knowledges, attitudes and behaviors

A questionnaire on HH, knowledges attitudes and behaviors was designed. The sample to which the questionnaire will be applied tol the professionals selected from both groups, after the first SO has been performed and before training, in the intervention group and in the usual clinical practice group at the same time.

The questionnaire consists of 17 questions: twelve with item on the Likert scale with answers that can be graded from 1 to 4, where 4 is the maximum value; four questions with multiple choice answers and one question with a single answer.

Of the total number of questions, nine refer to HH and eight questions refer to generic patient safety issues in order to "camouflage" the direct questions on HH.

##### Validation of the questionnaire

A pilot group of 25 healthcare workers from a health center will be provided to determine its reliability and validity.

Cronbach's alpha coefficient will be calculated for the scores of the professionals at two points in the questionnaire in order to validate the internal consistency and reliability. The questionnaire will be administered again to the pilot group after a 20-day interval and the intraclass correlation coefficient will be calculated, in order to determine the reproducibility or reliability of the test-retest.

The validity of the content will be evaluated in two ways:

First, a group of experts will valued the ability of the questionnaire to evaluate all the aspects to be measured. A factorial analysis of the main components - the Varimax rotation - will be carried out to analyze the construct vality (the level to which the instrument reflects the theory of the phenomenon or concept being measured). The adequacy of the factorial analysis will be tested by the Kaiser-Meyer-Olkin measure and Bartlett's test of sphericity.

The questionnaire will be sent by post to the professional, preceded by a generic informative letter (to maximize the response rate). A questionnaire with a more detailed introductory letter will be sent a few days later. A reminder will be sent ten days after sending, which will once again include the questionnaire and a note of thanks.

#### -"Professional burnout" questionnaire

The validated Spanish version of the "Maslach Burnout Inventory (MBI)" will be used [24]. This questionnaire has 22 item with seven response options (Likert scale from 0 to 6), measuring the three aspects of burnout: emotional exhaustion, depersonalization and personal accomplishment. Scores are obtained by totaling the values of the item, and each subscale is calculated separately. They are not combined, and a total MBI score is not obtained.

The MBI questionnaire will be applied with the questionnaire on knwoledges, attitudes and behaviors. Due to the sensitive nature of the questions, it is important that the professionals do not know that they are answering a questionnaire on professional stress. It will be presented as a scale of work attitudes.

### Intervention program

The HH training workshops will be carried out in the health centers assigned to the intervention group. A combined intervention strategy will be applied by:

1- Training in theoretical-practical workshops for the professionals healthcare in the intervention group on HH techniques. The strategy will be multi-faceted (many perspectives), multimodal (many procedures) and multidisciplinary.

The HH training workshops will be focused on strategies for creating changes in behaviors, beliefs and habits concerning traditional hygiene. There will also be an emphasis on morbidity, mortality, the costs related with NI and on the epidemiological evidence of the effects of a conclusive improvement in HH.

There will be a practical section to familiarize professionals with the ideal technique for achieving the maximum effectiveness in HH. Participatory techniques, group discussions and procedure demonstrations will be used.

2- The introduction of alcohol-based solutions, in all rooms in the intervention centers, for everyday use in healthcare.

They will be installed after the first SO and before the training workshop. Compared to traditional washing with soap and water, alcoholic products have been more effective in terms of reducing the pathogens load on the skin, having a longer residual effect and leading to less skin dryness [12,13,15,25].

3. "Workplace reminders" as a part of the multimodal strategy for promoting HH, including leaflets, posters and other materials placed at key points in the health centers in order to remind professionals that they must maintain regular and effective HH.

### Area of study

A multicenter study of 21 health centers in north-eastern Madrid from Spain with 600,000 inhabitants.

### Sample for study

- Target population.- Primary healthcare workers in Madrid (physicians, odontostomatologists, pediatricians, nurses, dental hygienists, midwife, and nursing auxiliaries) agreeing to participate.

- Exclusion criteria: Professionals that do not sign the informed consent.

### Sample size

#### Predetermination of the sample size

We estimated the required sample size a priori, assuming a power of 85% and an α

0,05. Our simple size was to detect a minimum difference between groups of five variation points (standard deviation 10 points) in scores on the HH variation compliance, giving and estimated sample size of 72 professionals in each group (intervention and usual clinical healthcare).

By assigning the intervention by center and assigning an average size to each center of 20 professionals, and considering an Intra-Center Correlation Coefficient (ICC) of 0.01, 10 health centers would be necessary - five for each group. Twenty professionals will be chosen randomly from each center, giving a final sample size of 100 professionals in each group.

The "Cluster Sample Size calculator" software package developed by the Health Services Research Unit of the University of Aberdeen (Scotland) and the ICC assumptions published for Primary Healthcare by Seuc AH et al (Rev Cubana Angiol y Cir Vasc 2001; 2(2): 117-22) were used for these calculations.

#### Selection of the sample

Multistage. First, 5 centers will be selected randomly for each group (intervention and usual clinical practice). Twenty professionals will then be selected in each center by stratified sampling for each type of healthcare profession. The randomization process is anticipated with the EPIDAT 3.0 statistics program.

### Data analysis

Statistical analysis will be carried out using the program SPSS v.15 (Chicago, Illinois)

- Descriptive analysis will be carried out with the median, standard deviation and minimum and maximum values. In asymmetrical distributions, the median will be used as a measure of centralization and the 25 and 75 percentiles as measures of dispersion. Confidence intervals of 95% will be calculated. The absolute and relative frequency will be given for the qualitative variables. A comparison table of the baseline characteristics of the differents intervention groups will be presented.

- A covariance analysis model (ACOVS), for the main objective, will be carried out for repeated measures. The dependent variable will be the variation of correct HH compliance in each observation period (1 and 6 months) compared to the baseline. The results of the questionnaire on knwoldges, attitudes, behaviors and patient safety will be expressed as relative frequencies, as with a median score on a scale from 1 to 4. The results will be shown with a confidence interval of 95%.

- A logistic regression analysis will be carried out, with a dependent variable that will be HH compliance and the main explanatory variable will be the type of intervention. The covariables for which it will be adjusted are: the variables which have shown an imbalance and those with a biological basis or which are potentially confusing in the comparison table of both groups, such as: age, years of employment experience, type of profession, type of contract, knowledges, attitudes and behaviors regarding HH, professional burnout. The manually controlled "Backstep LR" method will be used.

## Limitations of the study

The possible limitations are the lack of co-operation by professionals in the health centers, as they will not initially be informed about the objectives of the study, for they do not feel to be studied about their HH compliance (the Hawthorne effect), which could lead to the data collection being compromised. In that case, we would increase the size of the sample.

In order for the sample to be representative of all the professionals, there will be a random stratified selection of the professionals in each center, taking into account the proportion of each professional in the area study (43% physicians, 38.6% nurses, 10.3% pediatricians, 3.1% nursing auxiliaries, 1.6% dental hygienists, 1.6% midwifes).

A multivariate analysis (logistic regression) will be carried out to check for possible confusion factors that could distort o the real effect of the intervention on HH compliance.

Likewise, in order to reduce an incorrect classification, there will be only one observer.

## Ethical considerations

The study complies with the Helsinki Declaration and its subsequent revisions, and regulations of clinical best practice.

The study protocol has been approved by the Clinical Research Ethics Committee of the Hospital Ramón y Cajal in Madrid from Spain.

### Informed consent

The professionals will sign an informed consent declaration before the observation. As the observation will take place in consulting rooms, the patients will be informed of the presence of the observers in the room, as it is not ethical to allow the observer to be present in a confidential environment between the healthcare workers and patient without having informed the latter.

### Confidentiality of data

The researchers will respect the confidentiality of the study data and to ensure compliance with the Constitutional Law 15/1999 concerning the Protection of Personal Data.

## Discussion

The research team aims to evaluate hand hygiene compliance among healthcares workers to improve the quality and efficiency of the health services within the National Health System.

## List of abbreviations

WHO: World Health Organization; NI: Nosocomial Infection; HH: Hand Hygiene; HCWs: Hand Health Workers Centers; TP: Training Program; SO: Structured Observation; MBI: Maslach Burnout Inventory; ACOVS: A covariance analysis model.

## Competing interests

The authors declare that they have no competing interests.

## Authors' contributions

CMM is the Lead Researcher. She developed the design of the study and wrote the manuscript. AC contributed to the design of the study and its realization and reviewed the manuscript. MASF contributed to the design of the study and substantially to the statistical analysis and reviewed the manuscript. JCAH contributed to the design of the study and its realization and reviewed the manuscript. RAS, IGF, FEM coordinated the realization of the study. ECSP, SSD contributed to the design of the study. All the authors have approved the final version of the manuscript.

## Pre-publication history

The pre-publication history for this paper can be accessed here:

http://www.biomedcentral.com/1471-2458/9/469/prepub
